# Clinical Lectures on Paralysis

**Published:** 1855-09

**Authors:** 


### BIBLIOGRAPHICAL NOTICES.

*Clinical Lectures upon Paralysis, Disease of the Brain, and the Affections of the Nervous System.* By R. B. Todd, M. D., F. R. S. Philadelphia—Lindsay & Blackiston.

We have "line upon line" upon Cholera and its treatment, volume after volume upon fevers, monographs, essays and treatises upon malaria, contagion, &c., all valuable, and each in itself of grave import, but we have not recently met with a work more valuable than the above, not more in the abiding importance of the subject it treats than the able manner in which it is performed.

The more extensive our experience in cerebral diseases, the more we are inclined to condemn indiscriminate bleeding with the view to the control of cerebral phenomena. We have deemed it our duty, because of the position we have occupied, to enter our solemn caveat against an error which we believe has been often a fatal one. The author shows that cerebral symptoms are often the result of too small an amount of blood in the brain. Not only is this the case, but often that blood may be sadly defective in normal proportion. A diminution of the life—imparting constituents—the red corpuscles, will usually result in cerebral disturbance as in protracted cases of gastro-enteritis in children, and yet we have seen in these cases cupping and leeching, from the temples and nape of the neck, resorted to for their control; when stimulants are demanded and food is needed to furnish material from which to form the blood into its normal proportion of constituents. In this case there is a loss of sensorial power as in delirium tremens, and then again the blood in other cases acquires a poison, the depressing effect of which will disorder the brain as in typhoid fever, in which case such agents as will increase the biotic force will be indicated. So also in cases of insanity in which the nervous debility is the cause of the aberation. Irritation is usually the hand-maid of debility, in which case tonics and stimulants are often profitable in insanity.

This work exhibits a chasteness of style which with the valuable facts it contains, together with an intrinsic superiority every where manifest in its pages, constitutes it one of the most important additions to medical literature extant.

